# Media Reports as a Source for Monitoring Impact of Influenza on Hospital Care: Qualitative Content Analysis

**DOI:** 10.2196/14627

**Published:** 2020-03-04

**Authors:** Daphne F M Reukers, Sierk D Marbus, Hella Smit, Peter Schneeberger, Gé Donker, Wim van der Hoek, Arianne B van Gageldonk-Lafeber

**Affiliations:** 1 Centre for Infectious Disease Control National Institute for Public Health and the Environment Bilthoven Netherlands; 2 Regional Laboratory for Medical Microbiology and Infection Prevention Jeroen Bosch hospital 's-Hertogenbosch Netherlands; 3 Nivel Netherlands Institute for Health Services Research Utrecht Netherlands

**Keywords:** influenza, severe acute respiratory infections, SARI, surveillance, media reports, news articles, hospital care

## Abstract

**Background:**

The Netherlands, like most European countries, has a robust influenza surveillance system in primary care. However, there is a lack of real-time nationally representative data on hospital admissions for complications of influenza. Anecdotal information about hospital capacity problems during influenza epidemics can, therefore, not be substantiated.

**Objective:**

The aim of this study was to assess whether media reports could provide relevant information for estimating the impact of influenza on hospital capacity, in the absence of hospital surveillance data.

**Methods:**

Dutch news articles on influenza in hospitals during the influenza season (week 40 of 2017 until week 20 of 2018) were searched in a Web-based media monitoring program (Coosto). Trends in the number of weekly articles were compared with trends in 5 different influenza surveillance systems. A content analysis was performed on a selection of news articles, and information on the hospital, department, problem, and preventive or response measures was collected.

**Results:**

The trend in weekly news articles correlated significantly with the trends in all 5 surveillance systems, including severe acute respiratory infections (SARI) surveillance. However, the peak in all 5 surveillance systems preceded the peak in news articles. Content analysis showed hospitals (N=69) had major capacity problems (46/69, 67%), resulting in admission stops (9/46, 20%), postponement of nonurgent surgical procedures (29/46, 63%), or both (8/46, 17%). Only few hospitals reported the use of point-of-care testing (5/69, 7%) or a separate influenza ward (3/69, 4%) to accelerate clinical management, but most resorted to ad hoc crisis management (34/69, 49%).

**Conclusions:**

Media reports showed that the 2017/2018 influenza epidemic caused serious problems in hospitals throughout the country. However, because of the time lag in media reporting, it is not a suitable alternative for near real-time SARI surveillance. A robust SARI surveillance program is important to inform decision making.

## Introduction

### Surveillance of Severe Acute Respiratory Infections

The long and intense influenza epidemic in winter 2017/2018 in a number of European countries, including the Netherlands, led to substantial morbidity and increased mortality, especially because of pneumonia as a complication of influenza virus infection [1]. A sudden increase in the number of patients requiring hospitalization for (complications of) acute respiratory infections may pose a significant burden for hospitals in managing bed and staff capacity. It may also severely limit the possibilities to isolate patients suspected of influenza [2,3]. Surveillance of severe acute respiratory infections (SARI), defined as an acute respiratory infection requiring hospitalization, is considered a priority by the European Centre for Disease Prevention and Control, the World Health Organization, and individual countries. However, establishing surveillance systems in hospitals has proven difficult in many countries [4-6].

Ideally, surveillance of complications from influenza virus infection in sentinel hospitals would mimic the well-organized surveillance of influenza-like illness (ILI) or acute respiratory infections and influenza infection by sentinel general practitioners (GPs) in primary care [1]. However, in the European region, very few countries have established syndromic SARI surveillance in combination with testing for influenza virus [7]. In the Netherlands, limited real-time data on SARI are available from a pilot project in 2 hospitals [8], but this does not yet provide a nationally representative picture. Therefore, with a lack of national hospital surveillance data to guide health care measures, individual hospitals had to revert to ad hoc crisis management when patient numbers started to increase beyond capacity.

### Alternative Surveillance Methods

In the absence of nationally representative SARI surveillance, nontraditional Web-based data sources could improve the SARI surveillance. We could not identify previous reports in which media content was analyzed to assess impact on hospital capacity. However, in recent years, several studies have explored the use of alternative surveillance methods for influenza based on internet search terms. An example of internet-based surveillance system is Google Flu Trends, which monitors health-seeking behavior by using data on influenza-related searches to estimate the incidence of ILI in a specific region [9]. It has shown promising results during regular winter seasons [9]; however, it did not predict the 2009 influenza pandemic [10,11]. Multiple other internet-based surveillance systems have been developed over the years; however, it is often complex and cumbersome for epidemiologists to extract the relevant information from large amounts of data on social media or search engine queries [12]. Furthermore, media reports, in comparison with internet queries, have shown to contain more specific and official data in relation to influenza and other public health problems. In a study based on the 2009 pandemic, media reports were analyzed in relation to several influenza surveillance methods [11]. A study by Olayinka et al [13] showed that media reports were useful as a supplemental data source for the real-time mortality monitoring related to Hurricane Sandy. As the capacity problems that hospitals faced were reported in local, regional, and national media, these could potentially be a suitable supplemental data source to specifically assess the impact of hospitalized SARI patients during an influenza epidemic.

### Objectives

The aim of this study was to assess whether media reports during the 2017/2018 influenza epidemic provided relevant information for estimating the impact of influenza on hospital care as an indicator of the severity of the epidemic in the absence of traditional hospital-based epidemiological data.

## Methods

### Search Strategy for Media Reports

A search was conducted using Coosto (Coosto), which is a Web-based media monitoring and analytics program. The search term in Dutch *griep EN ziekenhuis* (in English: *flu/influenza AND hospital*) was used. There is only 1 word for flu/Influenza in Dutch used in media reporting, that is, *griep*. Only articles from the Netherlands published on regional or national news websites during the influenza season (week 40 of 2017 until week 20 of 2018) were selected. Trend in weekly number of news articles from the Coosto search was plotted against trends in the different influenza surveillance systems that were available on a weekly basis. A content analysis was performed on a selection of news articles with data on influenza in hospitals during the 2017/2018 influenza season. The relevance of the news articles and possible duplicates was only assessed for the content analysis.

### Available Respiratory Surveillance Systems in the Netherlands

#### Influenza-Like Illness

The basis for the weekly, near real-time surveillance of influenza in the Netherlands is the incidence of ILI as reported by approximately 40 GP sentinel practices participating in the Nivel Primary Care Database [14]. The population of these sentinel practices covers 0.7% of the Dutch population and is nationally representative for age, sex, regional distribution, and population density [15]. The ILI incidence is calculated as the number of patients with a new episode of ILI divided by the total number of enlisted patients in the participating sentinel GP practices. As all Dutch residents are registered in a general practice, the number of enlisted patients represents the general population. An influenza epidemic is declared when the ILI incidence is >5.1 per 10,000 inhabitants for a consecutive 2 weeks and when influenza virus is detected in swabs from ILI patients.

#### Community-Acquired Pneumonia in Primary Care

Pneumonia data are also obtained from Nivel Primary Care Database but from a larger group of GPs (approximately 400) based on automatic extraction of weekly number of patients consulting their GP for pneumonia (International Classification of Primary Care code R81) divided by the total number of enlisted patients in the participating GP practices [1].

#### Severe Acute Respiratory Infections

Data on SARI incidence is currently limited to information from 3 hospitals participating in a pilot SARI surveillance program. In our study, data from 1 hospital were used, the Jeroen Bosch hospital (JBH) in ‘s-Hertogenbosch, as these were the most robust data available. The SARI incidence was retrospectively based on a selection of financial codes that every Dutch hospital must use for reimbursement from health insurance companies related to the clinical syndrome SARI divided by the number of persons (approximately 323,000) in the catchment area of JBH [16].

#### Mortality Monitoring

In the Netherlands, all-cause deaths are notified to municipalities and then reported to Statistics Netherlands, which collects and monitors all Dutch vital statistics [1].

#### Virological Surveillance

Finally, on a weekly basis, about 19 Dutch virological laboratories report the number of positive diagnoses of several viral pathogens, including influenza. Details on the different surveillance systems for the 2017/2018 influenza season can be found in Reukers et al [1].

### Media Content Analysis

After the search in Coosto, 2 researchers separately scanned all titles for relevance. Articles were excluded if the title was unrelated to influenza in hospitals in the Netherlands or if it was a duplicate news article. Subsequently, full-text articles were assessed and excluded if irrelevant or duplicate. A qualitative content analysis was performed on the remaining articles. In each news article, the following information was identified: whether (1) the article was recently published, (2) it came from a designated spokesperson, (3) the name and place of the hospital was mentioned, (4) it was about a specific hospital department, and (5) the specific problems pertaining to influenza and the implemented preventive/response measures were mentioned. This was guided by the paper from Groeneveld et al who already listed the most common problems and prevention/response measures during the 2017/2018 influenza epidemic [17]. These were applied into the content analysis by categorizing and counting the number of problems and prevention/response measures reported by each hospital in the news articles. The problems reported by hospitals in the news articles were categorized as (1) hospital admission stops, (2) postponing nonurgent surgical procedures likely caused by the high number of influenza patients, (3) staff capacity problems because of influenza, and/or (4) other influenza epidemic-related problems [17]. Prevention or response measures were categorized as (1) ad hoc crisis management, (2) regional cooperation, (3) point-of-care testing (POCT), (4) cohort isolation for influenza patients, or (5) other prevention and control measures [17].

### Statistical Analysis

Using SAS version 9.4, Pearson correlation coefficients (significant at .05 level) were computed between the weekly number of media reports and the weekly ILI incidence, weekly number of pneumonia consultations per 10.000 inhabitants, weekly SARI incidence, weekly number of all-cause deaths, and weekly influenza diagnoses reported in the virological laboratory surveillance.

### Ethics

The Dutch Medical Research Involving Human Subjects Act (WMO) does not apply to this study, and therefore, an official approval by a Medical Ethical Research Committee is not required under the WMO. Furthermore, all data are publicly available. Surveillance data used in this study are available on the Nivel website (ILI incidence and pneumonia), the National Institute for Public Health and Environment (RIVM) website (SARI incidence and virological data), and the website of Statistics Netherlands [18].

## Results

### Comparisons of Media Reports With Surveillance Systems

**Figure 1:**
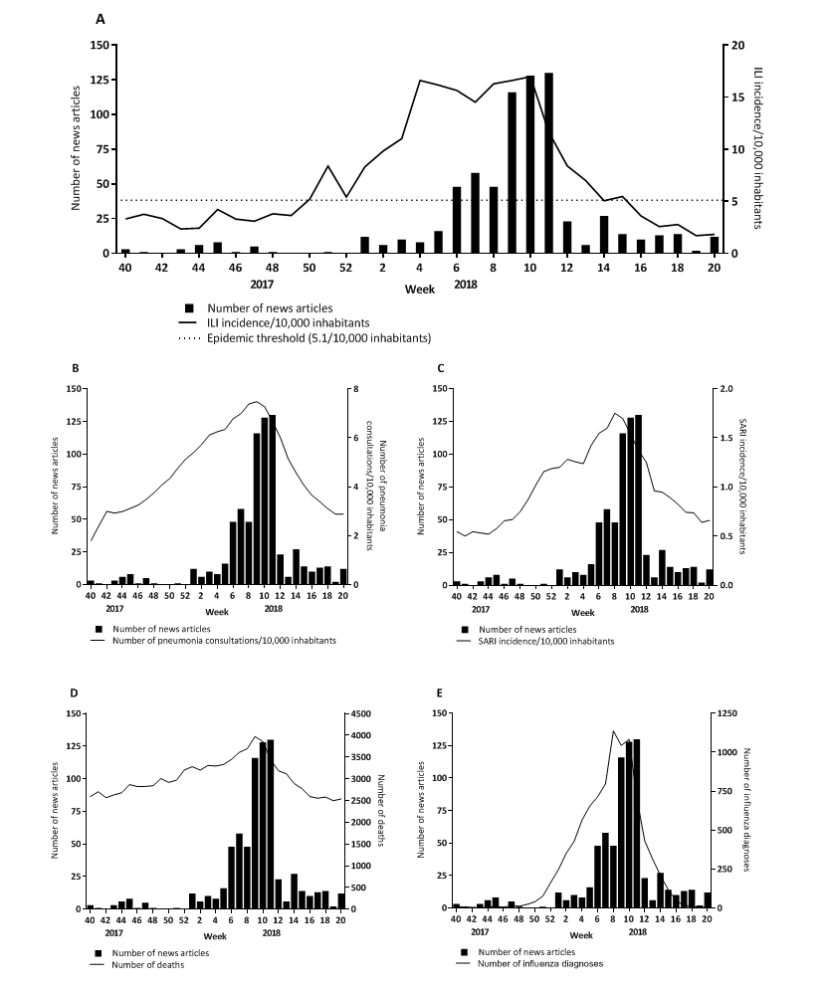
Weekly number of news articles and (A) influenza-like illness incidence per 10,000 inhabitants in general practitioner (GP) practices, (B) of patients consulting their GP for pneumonia per 10,000 inhabitants, (C) severe acute respiratory infections incidence per 10,000 inhabitants in Jeroen Bosch Hospital, (D) number of deaths, and (E) number of influenza diagnoses reported in the virological laboratory surveillance during the 2017/2018 influenza season in the Netherlands. ILI: influenza-like illness; SARI: severe acute respiratory infections.

The large majority of the weekly number of news articles from the Coosto search (n=730) during the 2017/2018 influenza season in the Netherlands coincided with the influenza epidemic as defined by the ILI incidence (Figure 1). The peak in ILI incidence preceded the peak in the number of articles by 5 weeks. The ILI incidence reached a peak in week 4 of 2018 and remained high until week 10 of 2018, while the media coverage increased later from around week 9 and reached a peak in week 11 of 2018. Both trends show a steep decrease after week 10 and 11, respectively. On visual inspection, similar trends were observed for the number of news articles in relation to weekly pneumonia consultations in primary care, SARI surveillance, all-cause mortality, and influenza laboratory diagnoses (Figure 1). The trends in ILI incidence and media reports are significantly correlated (Table 1). Correlations were even stronger for pneumonia in primary care, SARI in the JBH, influenza laboratory diagnoses, and all-cause mortality (Table 1).

**Table 1:** Correlation between the weekly number of news articles on influenza in hospitals and the weekly influenza-like illness incidence, pneumonia consultations, severe acute respiratory infections incidence, number of deaths, and influenza laboratory diagnoses.

Weekly number of news articles correlated with	Correlation coefficient	*P* value
Influenza-like illness incidence^a^	0.65	<.001
Number of pneumonia consultations^a^	0.67	<.001
Severe acute respiratory infections incidence^a^	0.77	<.001
Number of all-cause deaths	0.72	<.001
Number of influenza laboratory diagnoses^b^	0.79	<.001

^a^Per 10.000 inhabitants.

^b^Reported in the virological laboratory surveillance.

### Selection of Media Reports

**Figure 2:**
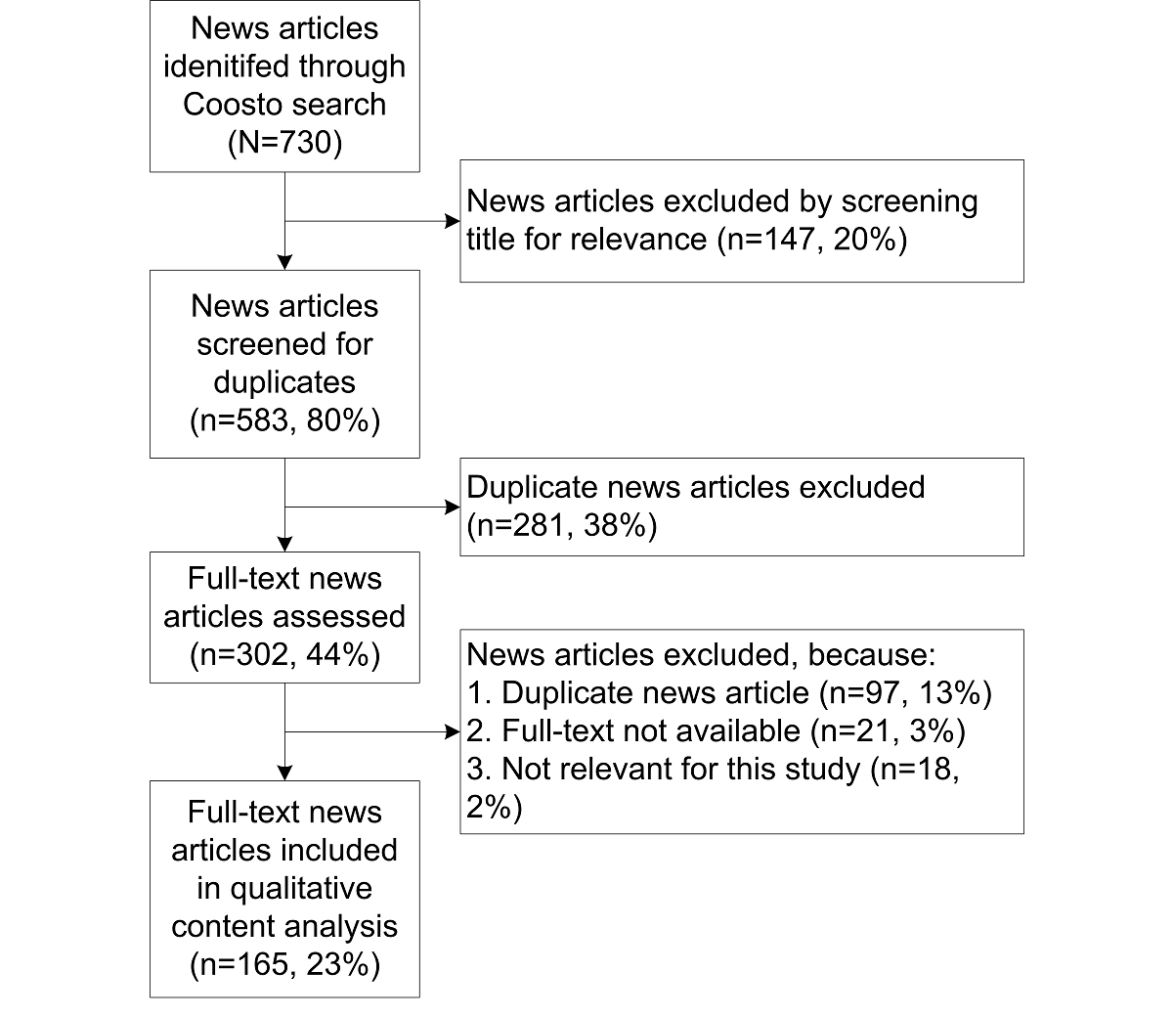
Flowchart presenting the inclusion and exclusion of regional or national Dutch news articles related to influenza in hospitals in the Netherlands during the 2017/2018 influenza season (week 40 of 2017 and until week 20 of 2018) from the Coosto database search using the term flu AND hospital.

For the content analysis, 147 of the 730 news articles were excluded based on the title (Figure 2). The remaining 583 articles were screened for duplicates, leaving 302 news articles to be assessed in full text. Ultimately, 165 (165/717, 23.0%) news articles were included in the qualitative content analysis (Figure 2).

### Content of Media Reports

The 165 news articles included 77 articles citing 1 or more specific hospitals or contained a statement from 1 or more hospitals, often by designated spokespersons. The other 88 news articles contained more general information about influenza and/or hospitals.

#### Geographical Distribution and Type of Reported Problems

**Figure 3:**
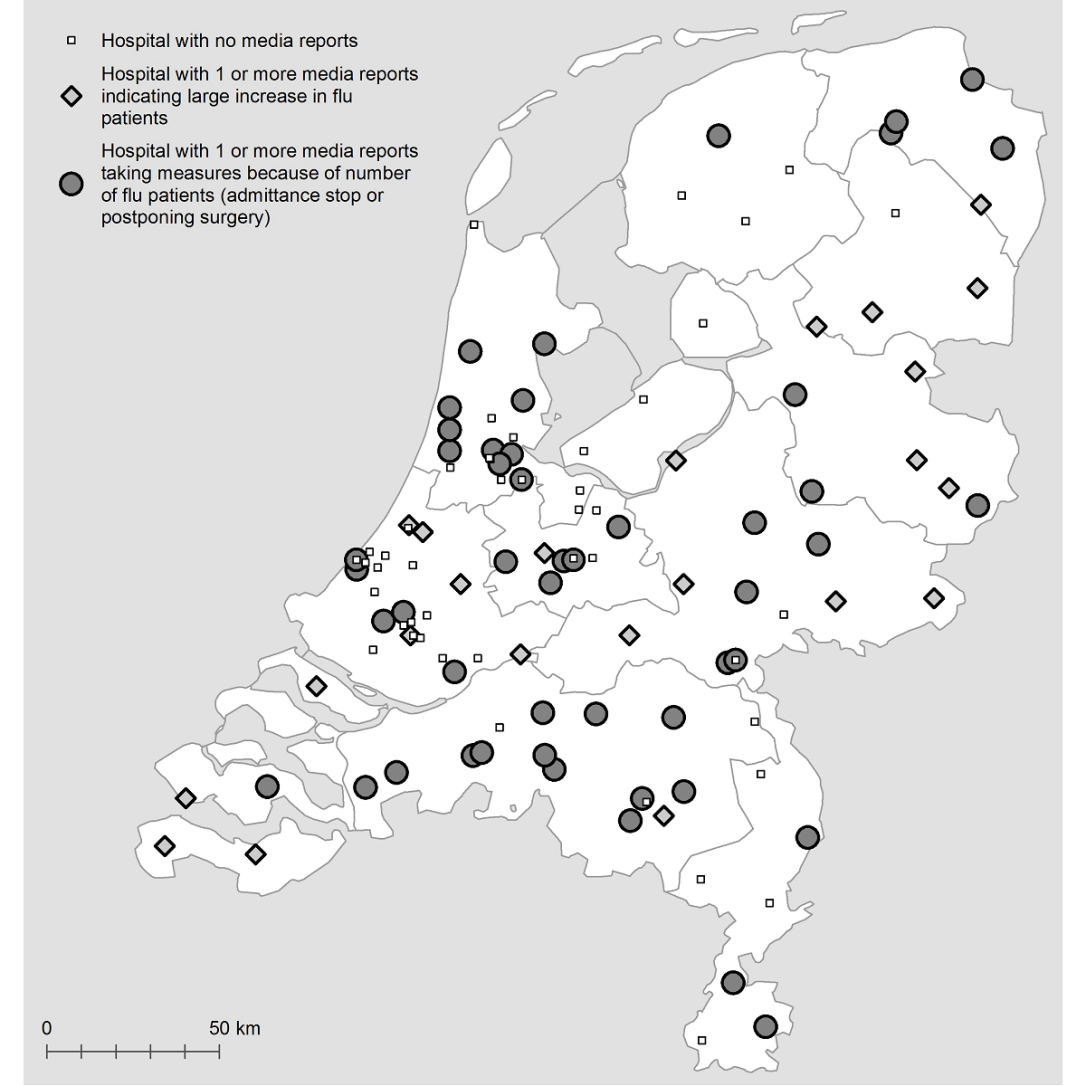
Map of the Netherlands with all hospital locations and type of media reports per hospital location.

Results from the content analysis show that 52 different hospital organizations were named in the included news articles with 69 hospital locations. These included 5 academic teaching hospitals, 1 children’s hospital, 35 top clinical teaching hospitals, and 28 general hospitals. These locations were spread across the Netherlands (Figure 3). Of the 69 hospital locations, 23 (33%) reported a large increase in influenza patients but were still able to cope with the number of admitted patients. The remaining 46 (46/69, 67%) hospitals had to take measures at least once during the influenza season; 29 (29/69, 63%) hospitals had to postpone nonurgent surgical procedures, 9 (9/69, 20%) instated a temporary admission stop, and 8 (8/69, 17%) had to take both of these measures (postponing surgery and admission stop). Of these 46 hospital locations, 20 (43%) indicated that the epidemic caused the largest problems in the emergency department (ED). A total of 25 of the 46 hospitals also indicated problems because of staff shortages towing to sick leave caused by influenza. Furthermore, 13 hospitals mentioned a stagnating flow of patients from the hospital to nursing homes. This concerned elderly patients for whom ongoing hospitalization was not medically indicated but who could not be discharged because of social reasons.

#### Media Reports on Response and Preventive Measures

Of all the 69 hospital locations, 29 (42%) mentioned no specific response or preventive measures; 34 (49%) hospitals implemented some form of crisis management, such as flexible deployment of staff (working overtime) and flexible bed occupancy; and 8 (12%) hospitals mentioned a regional cooperation between hospitals. Furthermore, 5 (5/69, 7%) hospitals used POCT to accelerate clinical management (Admiraal de Ruyter hospital in Goes and Vlissingen, Albert Schweitzer hospital in Dordrecht, Amphia hospital in Breda, and JBH in ‘s-Hertogenbosch), and 3 (3/69, 4%) hospitals set up a separate influenza ward to isolate the influenza patients (JBH in ‘s-Hertogenbosch, Sint Antonius hospital in Woerden, and Waterland hospital in Purmerend).

#### Media Reports on Mortality and Vaccination

In the 88 news articles containing general information about influenza and hospitals, an important topic (13/88, 15%) was the high mortality rates, especially among the elderly, related to the influenza epidemic. An influential Dutch senior organization (KBO-PCOB) called this a silent disaster and started lobbying for a “winter mortality plan” to be better prepared in the future. Furthermore, several articles (7/88, 8%) discussed the effectiveness of the influenza vaccine, the low vaccine uptake (especially among hospital staff), and the possibility of mandatory influenza vaccination of hospital and nursing home staff.

The remaining news articles (68/88, 77%) included news on regional cooperation among hospitals to cope with the influenza epidemic, the pressure on ambulance care, crisis exercise to prepare for a large influenza epidemic, respiratory syncytial virus in very young children compared with influenza, general staff capacity issues causing problems during a major (influenza) epidemic, or only included general information on influenza or the current influenza epidemic.

## Discussion

### Principal Findings

Even though this study showed that trends in media reports are not a suitable timely surveillance measure, they do provide relevant information on the impact of influenza on hospitals. Media reporting clearly showed the severity of the epidemic with a large number of hospitals having problems with capacity and staff shortages affecting patient care in terms of admission stops and postponement of nonurgent procedures. Media reports are suitable for retrospective analysis of the impact of an influenza epidemic on hospitals; however, real-time data are still necessary for preparedness and response.

### Implications of Study Results

On the basis of media reports, the 2017/2018 influenza season clearly had a big impact on Dutch hospitals throughout the country. It confirms that real-time data on influenza-related hospital admissions are needed at local, regional, and national level to inform decision making for preparedness and public health response. Improving SARI surveillance was also one of the recommendations of the Outbreak Management Team that was convened by the Dutch Centre for Infectious Disease Control in response to the severe influenza epidemic. In the absence of a sustainable and robust SARI surveillance program, retrospective analysis of media reports on hospitalized influenza patients offers useful information about the impact of an influenza epidemic. However, using media reports for near real-time surveillance of hospitalized influenza cases with respect to preparedness and emergency control is less suitable because of the longer time lag between detection of the event and published media reports compared with other available surveillance systems. This is in line with a study by de Lange et al [11] comparing traditional routine ILI surveillance with other systems of surveillance and trends in pandemic-related newspaper and television coverage and showed that the increase and peak in media coverage did not precede increases in ILI incidence.

### Health Care Interventions

In the Netherlands, there was a lack of real-time nationally representative data that could have guided hospital management in preparing and implementing mitigating action. No national guidelines were issued, in contrast to the United Kingdom, where hospitals were advised by the National Health Service to defer nonurgent operations [19]. Hospitals already participate in training programs on how to deal with emergency situations, such as an influenza pandemic or Ebola outbreak [20]. However, there seems to have been no concerted effort in dealing with the large influx of patients during the 2017/2018 influenza epidemic. When there is pressure on hospital capacity, it seems that hospitals refer to ad hoc crisis management. It shows that there is a need for influenza outbreak response plans for hospital preparedness in managing outbreaks of SARI. An important problem mentioned in the media reports by 13 hospitals was that elderly patients could often not be discharged because of the social situation of the patient or because no beds were available in nursing homes. Furthermore, many informal caregivers of these elderly patients were possibly unavailable because of influenza [17]. This is a growing problem with an ageing population and the prevailing policy that elderly persons should be encouraged to live independently at home as long as possible. Therefore, the pressure on informal caregivers and hospital care will likely continue to increase.

### Influenza Point-of-Care Testing

Most SARI patients admitted to a hospital are not routinely tested for influenza virus infection. Without national hospital guidelines on influenza diagnostics, influenza testing occurs mainly at the discretion of the treating physician. Even in intensive care units, only about half of such patients are tested [21]. Influenza POCT can be performed by nurses in the ED and are available with a turnaround time of 20 min. It was demonstrated that implementation of POCT in combination with a designated ward for influenza-positive patients during the 2017/2018 epidemic led to a marked reduction in length of hospital stay [2]. Such a policy may have additional benefits by a decrease in costs and unnecessary use of antibiotics [22].

### Limitations of the Study

A limitation of using media reports for surveillance purposes is the potential selectivity of media reporting and thereby, the introduction of selection bias. Especially, reporting on response measures is of concern because it is unclear whether the report was initiated by a specific health event or by the hospitals’ aim for media attention. Moreover, performing qualitative content analysis on media reports is time consuming, and the choice of items to be analyzed is still rather arbitrary. Developing an automated surveillance system would be better to extract information.

### Conclusions

This study showed that the 2017/2018 influenza epidemic caused serious problems affecting hospitals throughout the country. Media reports are not suitable for near real-time surveillance because of the longer time lag compared with other surveillance systems. This stresses the importance of a robust SARI surveillance program to inform decision making, which is especially important in seasons with high or long-lasting influenza activity.

## Abbreviations

ED: emergency department
GP: general practitioner
ILI: influenza-like illness
JBH: Jeroen Bosch hospital
POCT: point-of-care testing
SARI: severe acute respiratory infections
WMO: Medical Research Involving Human Subjects Act

## References

[1] Reukers DF, van Asten L, Brandsema PS, Dijkstra F, Donker G, van Gageldonk-Lafeber AB, Hooiveld M, de Lange MM, Marbus S, Teirlinck AC, Meijer A, van der Hoek W (2018). National Institute for Public Health and the Environment (RIVM).

[2] Lankelma JM, Hermans MH, Hazenberg EH, Macken T, Dautzenberg PL, Koeijvoets KC, Jaspers JW, van Gageldonk-Lafeber AB, Lutgens SP (2019). Implementation of point-of-care testing and a temporary influenza ward in a Dutch hospital. Neth J Med.

[3] Centers for Disease Control and Prevention (CDC) National Center for Immunization and Respiratory Diseases (NCIRD).

[4] van den Wijngaard CC, van Pelt W, Nagelkerke NJ, Kretzschmar M, Koopmans MP (2011). Evaluation of syndromic surveillance in the Netherlands: its added value and recommendations for implementation. Euro Surveill.

[5] World Health Organization (2013). Global epidemiological surveillance standards for influenza.

[6] European Center for Disease Prevention and Control (ECDC) (2009). Meeting report surveillance and studies in a pandemic. https://www.ecdc.europa.eu/sites/portal/files/media/en/publications/Publications/0908_MER_Surveillance_and_Studies_in_a_Pandemic_Meeting_Report.pdf.

[7] Meerhoff TJ, Simaku A, Ulqinaku D, Torosyan L, Gribkova N, Shimanovich V, Chakhunashvili G, Karseladze I, Yesmagambetova A, Kuatbayeva A, Nurmatov Z, Otorbaeva D, Lupulescu E, Popovici O, Smorodintseva E, Sominina A, Holubka O, Onyshchenko O, Brown CS, Gross D (2015). Surveillance for severe acute respiratory infections (SARI) in hospitals in the WHO European region - an exploratory analysis of risk factors for a severe outcome in influenza-positive SARI cases. BMC Infect Dis.

[8] Marbus SD, Oost JA, van der Hoek W, Meijer A, Polderman FN, de Jager CPC, Groeneveld GH, Schneeberger PM, van Gageldonk-Lafeber AB (2016). Ernstige acute luchtweginfecties: de ontbrekende bouwsteen in de surveillancepiramide. [Severe acute respiratory infections: the missing building block in the surveillance pyramid]. Ned Tijdschr Med Micrbiol.

[9] Ginsberg J, Mohebbi MH, Patel RS, Brammer L, Smolinski MS, Brilliant L (2009). Detecting influenza epidemics using search engine query data. Nature.

[10] Cook S, Conrad C, Fowlkes AL, Mohebbi MH (2011). Assessing Google flu trends performance in the United States during the 2009 influenza virus A (H1N1) pandemic. PLoS One.

[11] de Lange MM, Meijer A, Friesema IH, Donker GA, Koppeschaar CE, Hooiveld M, Ruigrok N, van der Hoek W (2013). Comparison of five influenza surveillance systems during the 2009 pandemic and their association with media attention. BMC Public Health.

[12] Velasco E, Agheneza T, Denecke K, Kirchner G, Eckmanns T (2014). Social media and internet-based data in global systems for public health surveillance: a systematic review. Milbank Q.

[13] Olayinka OO, Bayleyegn TM, Noe RS, Lewis LS, Arrisi V, Wolkin AF (2017). Evaluation of real-time mortality surveillance based on media reports. Disaster Med Public Health Prep.

[14] (2016). Verheij RA, Koppes LLJ.

[15] Donker GA (2016). NIVEL Primary Care Database.

[16] Dutch Hospital Data.

[17] Groeneveld GH, Spaan WJ, van der Hoek W, van Dissel JT (2018). [The severe flu season of 2017-2018: making a case for the vaccination of healthcare professionals]. Ned Tijdschr Geneeskd.

[18] Netherlands Institute for Health Services Research (Nivel), National Institute for Public Health and the Environment (RIVM), Statistics Netherlands (CBS).

[19] (2018). NHS England.

[20] Adeoti AO, Marbus S (2018). The European Respiratory Society course on acute respiratory pandemics: how to plan for and manage them. ERJ Open Res.

[21] van Someren Gréve F, Ong DS, Cremer OL, Bonten MJ, Bos LD, de Jong MD, Schultz MJ, Juffermans NP, MARS consortium (2016). Clinical practice of respiratory virus diagnostics in critically ill patients with a suspected pneumonia: a prospective observational study. J Clin Virol.

[22] Dautzenberg P, Macken T, van Gageldonk-Lafeber AB, Lutgens S (2018). [Wave of flu under control with hospital-wide approach: Jeroen Bosch Hospital manages capacity problems.]. Medisch Contact.

